# Supplementary material for: Occurrence of Rickettsia spp., Hantaviridae, Bartonella spp. and Leptospira spp. in European Moles (Talpa europaea) from the Netherlands
Source: Microorganisms. 2022 Dec 22;11(1):41. doi: 10.3390/microorganisms11010041 (PMC9861085; doi:10.3390/microorganisms11010041)
Supplement: Supplementary file 1 [file microorganisms-11-00041-s001.zip › microorganisms-2100140-supplementary.pdf]

Supplementary material for research article:

Occurrence of *Rickettsia* spp., *Hantaviridae*, *Bartonella* spp. and *Leptospira* spp. in European moles (*Talpa europaea*) from the Netherlands

Table S1: Primers used in this study

Pathogen	Target gene	Primer	Primer sequence (5'-3')	Reference	Tissue used for detection
Hantaviridae	L-segment	F1	ATGTAYGTBAGTGCWGATGC	Klempa et al. (2006)	Lung
		R1	AACCADTCWGTYCCRTCAT		
		F2	TGCWGATGCHACIAARTGGTC		
		R2	GCRTCRTCWGARTGRTGDGCAA		
Leptospira spp.	secY	F	GCGATTCAGTTTAATCCTGC	Ahmed et al. (2009)	Kidney
		R	GAGTTAGAGCTCAAATCTAAG		
Bartonella spp. (detection)	ssra	F	GCTATGGTAATAAATGGACAATGAAATAA	Diaz et al. (2012)	Spleen
		R	GCTTCTGTTGCCAGGTG		
		Probe	ACCCCGCTTAAACCTGCGACG		
Bartonella spp. (sequencing)	gltA	F	GGGGACCAGCTCATGGTGG	Norman et al. (1995)	Spleen
		R	ATTGCAAAAGAACAGTAAACA		
Spotted fever group *Rickettsia*	Rstenos	F	TCGCAAATGTTCACGGTACTTT	Stenos et al. (2005)	Spleen
		R	TCGTGCATTTCTTTCCATTGTG		
		Probe	TGCAATAGCAAGAACCGTAGGCTGGATG		
Anaplasma phagocytophilum	Msp2	F	ATGGAAGGTAGTGTTGGTTATGGTATT	Courtney et al. (2004)	Spleen
		R	TTGGTCTTGAAGCGCTCGTA		
		Probe	TGGTGCCAGGGTTGAGCTTGAGATTG		
Babesia spp.	18S	F	CAGCTTGACGGTAGGGTATTGG	Øines et al. (2012)	Spleen
		R	TCGAACCCTAATTCCCCGTTA		
		Probe	CGAGGCAGCAACGG		
Babesia microti	ITS	F	CTCACACAACGATGAAGGACGCA	Azagi et al. (2021)	Spleen
		R	AACAGAGGCAGTGTGTACAATACATTCAGA		
		Probe	GCA+GAATTTAG+CAAAT+CAACAGG		
Francisella tularensis	FopA	F	ATCTAGCAGGTCAAGCAACAGGT	Versage et al. (2003)	Spleen
		R	GTCAACACTTGCTTGAACATTTCTAGATA		
		Probe	CAAACTTAAGACCACCACCCACATCCCAA		
Borrelia burgdorferi	OspA	F	AATATTTATTGGGAATAGGTCTAA	Heylen et al. (2013)	Spleen
		R	CTTTGTCTTTTTCTTTRCTTACAAG		
		Probe	AAGCAAAATGTTAGCAGCCTTGA		
Borrelia miyamotoi	flagellin	F	AGAAGGTGCTCAAGCAG	Hovius et al. (2013)	Spleen
		R	TCGATCTTTGAAAGTGACATAT		
		Probe	AGCACAACAGGAGGGAGTTCAAGC		
Neoehrlichia mikurensis	groE	F	CCTTGAAAATATAGCAAGATCAGGTAG	Jahfari et al. (2012)	Spleen
		R	CCACCACGTAACTTATTTAGTACTAAAG		
		Probe	CCTCTACTAATTATTGCTGAAGATGTAGAAGGTGAAGC		
Spiroplasma spp.	rpoB	F	TGTTGGACCAAACGAAGTTG	Krawczyk et al. (2020)	Spleen
		R	CCAACAATTGGTGTTTGGGG		
		Probe	GCTAACCGTGCTTTAATGGG		

Table S2: Moles tested in this study

Number	Location	Sex	Weight (gram)	*Leptospira* spp.	*Bartonella* spp.	*Hantaviridae* *	Spotted fever group *Rickettsia*
18-2292	Urk	F	93	-	-	-	-
18-2293	Urk	M	127	-	-	+	-
18-2294	Urk	F	77	-	-	-	-
18-2295	Urk	M	121	-	-	-	-
18-2296	Urk	F	103	-	+	-	-
18-2297	Urk	F	106	*Leptospira interrogans*	-	-	-
18-2298	Urk	F	93	-	-	+	-
18-2299	Urk	M	130	-	-	-	-
18-2300	Urk	M	137	-	+	+	-
18-2301	Urk	M	154	-	-	-	-
18-2302	Urk	M	116	-	+	+	-
18-2303	Urk	F	91	-	-	+	-
18-2304	Urk	F	97	-	+	+	-
18-2305	Urk	F	90	-	-	-	-
18-2306	Urk	F	108	-	-	+	-
18-2307	Urk	F	93	-	-	+	-
18-2308	Urk	F	93	-	-		-
18-2309	Urk	F	100	-	-	-	-
18-2310	Urk	F	94	-	+	+	-
18-2311	Urk	M	147	-	-	+	-
18-2312	Urk	F	76	-	-	-	-
18-2313	Urk	F	84	-	-	-	-
18-2314	Urk	F	103	-	-	-	-
18-2315	Urk	F	96	-	-	-	-
18-2316	Urk	F	73	*Leptospira kirschneri*	-	-	-
18-2317	Urk	F	86	-	-	+	-
18-2318	Urk	F	99	-	-	+	-
18-2319	Urk	F	94	-	-	+	-
18-2320	Urk	F	79	-	-	+	-
18-2321	Urk	F	95	-	-	+	-
18-2322	Urk	F	84	-	-	+	-
18-2323	Urk	M	122	-	+	+	-
18-2324	Urk	F	104	-	+	+	-
18-2325	Urk	M	118	-	-	-	-
18-2326	Urk	M	135	-	-	-	-
18-2327	Urk	M	122	-	-	-	-
18-2328	Urk	M	138	-	-	-	-
18-2329	Urk	M	137	-	-	-	-
18-2330	Urk	M	139	-	-	+	-
18-2331	Urk	M	125	-	+	-	-
18-2332	Urk	F	89	-	+	-	-
18-2333	Urk	F	93	-	-	-	-
18-2334	Urk	M	118	-	+	+	-

18-2335	Urk	F	87	-	-	+	-
18-2336	Urk	F	94	-	+	+	-
18-2337	Urk	F	87	-	+	-	-
18-2338	Urk	F	84	-	-	+	-
18-2339	Urk	F	89	-	-	+	-
18-2340	Urk	F	85	-	+	+	-
18-2341	Urk	M	129	-	-	+	-
18-2342	Urk	M	116	-	-	+	-
18-2343	Urk	F	82	-	+	+	-
18-2344	Urk	F	90	-	-	-	-
18-2345	Urk	F	86	-	-	+	-
18-2346	Urk	F	86	-	+	+	-
18-2347	Urk	F	90	-	-	-	-
18-2348	Urk	M	150	-	-	-	-
18-2349	Urk	M	143	-	-	-	-
18-2350	Urk	M	138	-	-	-	-
18-2351	Urk	F	103	-	-	+	-
18-2372	Urk	F	92	-	-	+	-
18-2373	Urk	F	84	-	-	-	-
18-2374	Urk	F	98	-	-	-	-
18-2375	Urk	F	98	-	+	-	-
18-2376	Urk	M	106	-	-	+	-
18-2377	Urk	F	91	-	-	+	-
18-2378	Urk	F	89	-	-	-	-
18-2379	Urk	F	98	-	-	-	-
18-2380	Urk	M	137	-	-	-	-
18-2381	Urk	F	87	-	-	-	-
18-2382	Urk	M	107	-	-	-	-
18-2383	Urk	F	96	-	-	+	-
18-2384	Urk	F	108	-	-	-	-
18-2385	Urk	M	129	-	-	-	-
18-2386	Urk	M	146	-	-	+	-
18-2387	Urk	M	140	-	-	+	-
18-2388	Urk	M	134	-	+	+	-
18-2389	Urk	M	160	-	-	-	-
18-2390	Urk	F	91	-	+	-	-
18-2421	Urk	F	89	-	-	-	-
18-2422	Urk	F	95	-	-	-	-
18-2423	Urk	F	87	-	+	-	-
18-2424	Urk	F	81	-	-	+	-
18-2425	Urk	F	83	-	-	-	-
18-2426	Urk	F	98	-	-	-	-
18-2427	Urk	F	95	-	-	-	-
18-2428	Urk	F	93	-	-	-	-
18-2429	Urk	F	81	-	-	+	-
18-2430	Urk	M	144	-	-	+	-
18-2431	Urk	F	99	-	-	-	-
18-2432	Urk	F	93	-	+	-	-
18-2433	Urk	M	113	-	-	-	-
18-2434	Urk	M	132	-	+	+	-

18-2435	Urk	F	91	-	-	+	-
18-2436	Urk	F	99	-	+	+	-
18-2437	Urk	M	140	-	+	-	-
18-2438	Urk	M	126	-	-	-	-
18-2439	Urk	M	148	-	+	-	-
18-2440	Urk	M	144	-	-	+	-
18-2441	Urk	F	104	-	+	-	-
18-2442	Urk	F	89	-	-	-	-
18-2443	Urk	F	95	-	-	-	-
18-2444	Urk	M	134	-	+	-	-
18-2445	Urk	M	159	-	-	-	-
18-2446	Urk	M	137	-	-	+	-
18-2496	Urk	F	95	-	+	-	-
18-2497	Urk	F	105	-	+	+	-
18-2498	Urk	F	101	-	-	+	-
18-2499	Urk	M	119	-	+	+	-
18-2500	Urk	M	137	*Leptospira interrogans*	+	+	-
18-2501	Urk	F	103	-	+	+	-
18-2502	Urk	M	149	-	-	+	-
18-2503	Urk	M	133	-	+	+	-
18-2504	Urk	M	131	-	+	+	-
18-2505	Urk	F	97	-	+	+	-
18-2506	Urk	F	91	-	-	-	-
18-2507	Urk	M	167	-	+	-	-
18-2508	Urk	F	95	-	+	+	-
18-2509	Urk	F	89	-	+	+	-
18-2510	Urk	F	81	-	+	+	-
18-2511	Urk	F	95	-	-	-	-
18-2512	Urk	F	95	-	+	+	-
18-2513	Urk	M	139	-	+	-	-
18-2514	Urk	F	91	-	+	+	-
18-2515	Urk	F	87	-	+	-	-
19-2693	Bergen op Zoom	M	120	-	+	+	-
19-2694	Bergen op Zoom	M	130	-	+	+	-
19-2695	Bergen op Zoom	M	88	-	-	-	-
19-2696	Bergen op Zoom	F	84	-	+	+	-
19-2697	Bergen op Zoom	M	n.d	-	+	+	-
19-2698	Bergen op Zoom	M	n.d	-	-	-	-
19-2699	Bergen op Zoom	F	102	-	+	-	-
19-2700	Bergen op Zoom	F	80	-	+	-	-
19-2701	Bergen op Zoom	F	74	-	+	-	-
19-2702	Bergen op Zoom	M	88	-	+	-	-
19-2703	Bergen op Zoom	M	114	-	+	+	-
19-2704	Bergen op Zoom	F	98	-	+	+	-
19-2705	Bergen op Zoom	F	86	-	-	+	-
19-2706	Bergen op Zoom	F	86	-	-	-	-
19-2707	Bergen op Zoom	M	122	-	+	-	-
19-2708	Bergen op Zoom	M	12	-	+	-	-
19-2709	Bergen op Zoom	F	74	-	+	+	-

19-2710	Bergen op Zoom	M	110	-	+	-	-
19-2711	Bergen op Zoom	F	78	-	+	-	-
19-2712	Bergen op Zoom	M	122	-	+	+	-
19-2713	Lage Zwaluwe	F	86	-	-	+	-
19-2714	Lage Zwaluwe	F	86	-	-	-	-
19-2715	Lage Zwaluwe	M	110	-	-	-	-
19-2716	Lage Zwaluwe	F	70	-	-	-	-
19-2717	Lage Zwaluwe	M	122	-	-	-	-
19-2718	Lage Zwaluwe	M	128	-	-	-	-
19-2719	Lage Zwaluwe	M	130	-	+	-	-
19-2720	Lage Zwaluwe	M	104	-	-	-	-
19-2721	Lage Zwaluwe	M	82	-	-	+	-
19-2722	Lage Zwaluwe	F	78	-	-	+	-
19-2723	Lage Zwaluwe	M	128	-	-	-	-
19-2724	Lage Zwaluwe	M	88	-	-	+	-
19-2725	Lage Zwaluwe	F	92	-	-	-	-
19-2726	Lage Zwaluwe	F	88	-	-	+	-
19-2727	Lage Zwaluwe	F	102	-	+	-	-
19-2728	Lage Zwaluwe	M	122	-	-	+	-
19-2729	Lage Zwaluwe	M	102	-	-	+	-
19-2730	Lage Zwaluwe	M	106	-	-	-	-
19-2731	Lage Zwaluwe	M	126	-	+	+	+
19-2732	Lage Zwaluwe	n.d.	104	-	-	+	-
19-2733	Schimmert	M	98	-	+	-	-
19-2734	Schimmert	M	114	-	-	+	-
19-2735	Schimmert	M	88	-	+	-	-
19-2736	Gennep	F	80	-	+	+	-
19-2737	Gennep	M	102	-	-	-	-
19-2738	Gennep	F	80	-	+	+	-
19-2739	Gennep	M	110	-	+	+	-
19-2740	Gennep	M	110	-	-	-	-
19-2741	Gennep	F	88	-	-	+	-
19-2742	Gennep	F	70	-	+	-	-
19-2743	Gennep	M	110	-	-	-	-
19-2744	Gennep	M	126	-	-	-	-
19-2745	Gennep	F	80	-	+	**+**	-
19-2746	Gennep	F	72	-	-	-	-
19-2747	Gennep	M	106	-	+	+	-

* All detected hantaviruses concern NVAV, only the one animal in bold (19-2745) indicates the animal in which BRGV was detected.

References

Ahmed, A., Engelberts, M. F., Boer, K. R., Ahmed, N., & Hartskeerl, R. A. (2009). Development and validation of a real-time PCR for detection of pathogenic *Leptospira* species in clinical materials. *PLoS One*, *4*(9), e7093. https://doi.org/10.1371/journal.pone.0007093

Azagi, T., Jaarsma, R. I., Docters van Leeuwen, A., Fonville, M., Maas, M., Franssen, F. F. J., Kik, M., Rijks, J. M., Montizaan, M. G., Groenevelt, M., Hoyer, M., Esser, H. J., Krawczyk, A. I., Modrý, D., Sprong, H., & Demir, S. (2021). Circulation of *Babesia* Species and Their Exposure to Humans through *Ixodes Ricinus*. *Pathogens*, *10*(4). https://doi.org/10.3390/pathogens10040386

Courtney, J. W., Kostelnik, L. M., Zeidner, N. S., & Massung, R. F. (2004). Multiplex real-time PCR for detection of *Anaplasma phagocytophilum* and *Borrelia burgdorferi*. *J Clin Microbiol*, *42*(7), 3164-3168. https://doi.org/10.1128/jcm.42.7.3164-3168.2004

Diaz, M. H., Bai, Y., Malania, L., Winchell, J. M., & Kosoy, M. Y. (2012). Development of a novel genus-specific real-time PCR assay for detection and differentiation of *Bartonella* species and genotypes. *J Clin Microbiol*, *50*(5), 1645-1649. https://doi.org/10.1128/jcm.06621-11

Heylen, D., Tijsse, E., Fonville, M., Matthysen, E., & Sprong, H. (2013). Transmission dynamics of *Borrelia burgdorferi* s.l. in a bird tick community. *Environ Microbiol*, *15*(2), 663-673. https://doi.org/10.1111/1462-2920.12059

Hovius, J. W., de Wever, B., Sohne, M., Brouwer, M. C., Coumou, J., Wagemakers, A., Oei, A., Knol, H., Narasimhan, S., Hodiamont, C. J., Jahfari, S., Pals, S. T., Horlings, H. M., Fikrig, E., Sprong, H., & van Oers, M. H. (2013). A case of meningoencephalitis by the relapsing fever spirochaete *Borrelia miyamotoi* in Europe. *Lancet*, *382*(9892), 658. https://doi.org/10.1016/s0140-6736(13)61644-x

Jahfari, S., Fonville, M., Hengeveld, P., Reusken, C., Scholte, E. J., Takken, W., Heyman, P., Medlock, J. M., Heylen, D., Kleve, J., & Sprong, H. (2012). Prevalence of *Neoehrlichia mikurensis* in ticks and rodents from North-west Europe. *Parasit Vectors*, *5*, 74. https://doi.org/10.1186/1756-3305-5-74

Klempa, B., Fichet-Calvet, E., Lecompte, E., Auste, B., Aniskin, V., Meisel, H., Denys, C., Koivogui, L., ter Meulen, J., & Krüger, D. H. (2006). Hantavirus in African wood mouse, Guinea. *Emerg Infect Dis*, *12*(5), 838-840. https://doi.org/10.3201/eid1205.051487

Krawczyk, A. I., van Duijvendijk, G. L. A., Swart, A., Heylen, D., Jaarsma, R. I., Jacobs, F. H. H., Fonville, M., Sprong, H., & Takken, W. (2020). Effect of rodent density on tick and tick-borne pathogen populations: consequences for infectious disease risk. *Parasit Vectors*, *13*(1), 34. https://doi.org/10.1186/s13071-020-3902-0

Norman, A. F., Regnery, R., Jameson, P., Greene, C., & Krause, D. C. (1995). Differentiation of *Bartonella*-like isolates at the species level by PCR-restriction fragment length polymorphism in the citrate synthase gene. *Journal of Clinical Microbiology*, *33*(7), 1797-1803. https://doi.org/doi:10.1128/jcm.33.7.1797-1803.1995

Øines, Ø., Radzijevskaja, J., Paulauskas, A., & Rosef, O. (2012). Prevalence and diversity of *Babesia* spp. in questing Ixodes ricinus ticks from Norway. *Parasit Vectors*, *5*, 156. https://doi.org/10.1186/1756-3305-5-156

Stenos, J., Graves, S. R., & Unsworth, N. B. (2005). A highly sensitive and specific real-time PCR assay for the detection of spotted fever and typhus group Rickettsiae. *Am J Trop Med Hyg*, *73*(6), 1083-1085.

Versage, J. L., Severin, D. D., Chu, M. C., & Petersen, J. M. (2003). Development of a multitarget real-time TaqMan PCR assay for enhanced detection of *Francisella tularensis* in complex specimens. *J Clin Microbiol*, *41*(12), 5492-5499. https://doi.org/10.1128/jcm.41.12.5492-5499.2003
